# A Method for Chlorophyll-a and Suspended Solids Prediction through Remote Sensing and Machine Learning †

**DOI:** 10.3390/s20072125

**Published:** 2020-04-09

**Authors:** Lucas Silveira Kupssinskü, Tainá Thomassim Guimarães, Eniuce Menezes de Souza, Daniel C. Zanotta, Mauricio Roberto Veronez, Luiz Gonzaga, Frederico Fábio Mauad

**Affiliations:** 1 Vizlab | X-Reality and Geoinformatics Lab, Graduate Programme in Applied Computing, Unisinos University, São Leopoldo 93022-750, Brazil; tainat@edu.unisinos.br (T.T.G.); dzanotta@edu.unisinos.br (D.C.Z.); VERONEZ@unisinos.br (M.R.V.);; 2 Department of Statistics, State University of Maringá-PR, Maringá 87020-900, Brazil; emsouza@uem.br; 3 São Carlos Engineering School, São Carlos 13566-590, Brazil; mauadffm@sc.usp.br

**Keywords:** chlorophyll-a, total suspended solids, remote sensing, machine learning, artificial neural networks, random forest, K nearest neighbors, water quality

## Abstract

Total Suspended Solids (TSS) and chlorophyll-a concentration are two critical parameters to monitor water quality. Since directly collecting samples for laboratory analysis can be expensive, this paper presents a methodology to estimate this information through remote sensing and Machine Learning (ML) techniques. TSS and chlorophyll-a are optically active components, therefore enabling measurement by remote sensing. Two study cases in distinct water bodies are performed, and those cases use different spatial resolution data from Sentinel-2 spectral images and unmanned aerial vehicles together with laboratory analysis data. In consonance with the methodology, supervised ML algorithms are trained to predict the concentration of TSS and chlorophyll-a. The predictions are evaluated separately in both study areas, where both TSS and chlorophyll-a models achieved R-squared values above 0.8.

## 1. Introduction

The preservation of water resources creates many global-scale challenges. Constant and dynamic monitoring of these environments is only viable through extensive use of technologies that allow inexpensive and effective monitoring [1]. Considering that the prediction of water quality parameters is a critical aspect in any aquatic system, the research on methods that allow this estimate in lakes and reservoirs has significant value [2].

Eutrophication is a process that leads to water deterioration in lentic environments (water is still and not rapidly moving), and this happens when a body of water becomes overly enriched with nutrients, mostly caused by anthropogenic activities, which induce excessive growth of algae in the environment. Chlorophyll-a, turbidity, and suspended solids are physicochemical water parameters related to eutrophication, and their evaluation can be used to assess the eutrophic state of water bodies [3]. Moreover, the suspended solids may indicate erosive processes within the watershed and additional water pollution since it can carry or store pollutants [4].

Generally, once the samples get to a laboratory, their concentrations are analyzed, and manual measurements are taken to extract these parameters. The involved processes are costly, time-consuming, and require skilled personnel [5]. Although there are accurate evaluations, they do not indicate the individual spatio-temporal variation of water quality [6].

In this sense, applications involving remote sensing can overcome the drawbacks of conventional monitoring since they allow real-time, spatial, continuous, and long-term monitoring in large areas [7,8]. The presence of components alters the spectral characteristics of the pure water, called Optically Active Components (OAC). The main OACs in water bodies are chlorophyll, suspended solids, and organic material [4]. The possibility of monitoring non-accessible areas and composing time series of water quality from historical data of remote sensing [9] are also factors that make this tool essential and its use promising.

Among the most common satellites in OAC remote sensing studies are Landsat 8 and Sentinel-2. Although Landsat is more widely used, the European Sentinel-2 satellite has promising characteristics: it has higher spatial resolution than Landsat 8 and also spectral bands in the region called the red edge, which is of great interest for water and vegetation studies.

Remote sensing serves as a powerful technique for monitoring environmental and seasonal changes, and its ability to remotely monitor water resources has increased in recent decades because of the quality and availability of satellite imagery data [10]. Even so, the analysis of small water bodies may not be adequate due to the medium image resolution of the most typical commercial satellites [8,11].

The combination of aerial image data obtained by Unmanned Aerial Vehicles (UAVs) together with modern pattern recognition systems is promising due to many factors: it has the potential to capture more details due to its higher spatial resolution; it can allow shorter revisit time, ensuring the possibility of constant and dynamic monitoring; it enables monitoring of hard-to-reach areas; it has an affordable cost compared to usual water collection and analysis methodologies; and finally, image acquisition is not affected by cloud cover, which may be a major limitation in the use of satellite imagery [8,12,13].

Another aspect to be considered in modeling water physicochemical variables through remote sensing is that the spatial distribution and time evolution of these parameters in ecosystems can be very complex and nonlinear [2] and that spatial and temporal trends can be difficult to understand at larger scales [11].

Due to the complex nature of aquatic environments, approaches involving learning algorithms are being used to improve the accuracy and reliability of predictive models generated by empirical methods [7,14,15,16,17]. Those learning algorithms, often called learners, can overcome traditional regression modeling difficulties to model non-linear relations between dependent and independent variables [18]. Machine Learning (ML) is the branch of artificial intelligence that aims to develop these algorithms that solve problems by consuming data and by approximation of functions that describe the behavior of the monitored phenomenon [19].

The purpose of this paper is to expand the work on [20], proposing a methodology to estimate chlorophyll-a and Total Suspended Solids (TSS) in water bodies through remote sensing data. The proposed framework is designed for different spatial resolutions, using data either from satellites or UAVs, depending on the required application or the size of the study area. Study cases on two distinct study areas are performed to show evidence of the applicability of the method. Both regions were visited in fieldwork campaigns to collect spatialized water samples providing data for several trials of different ML techniques. The next sections present those methodological steps and results.

## 2. Materials and Methods

**Figure 1:**
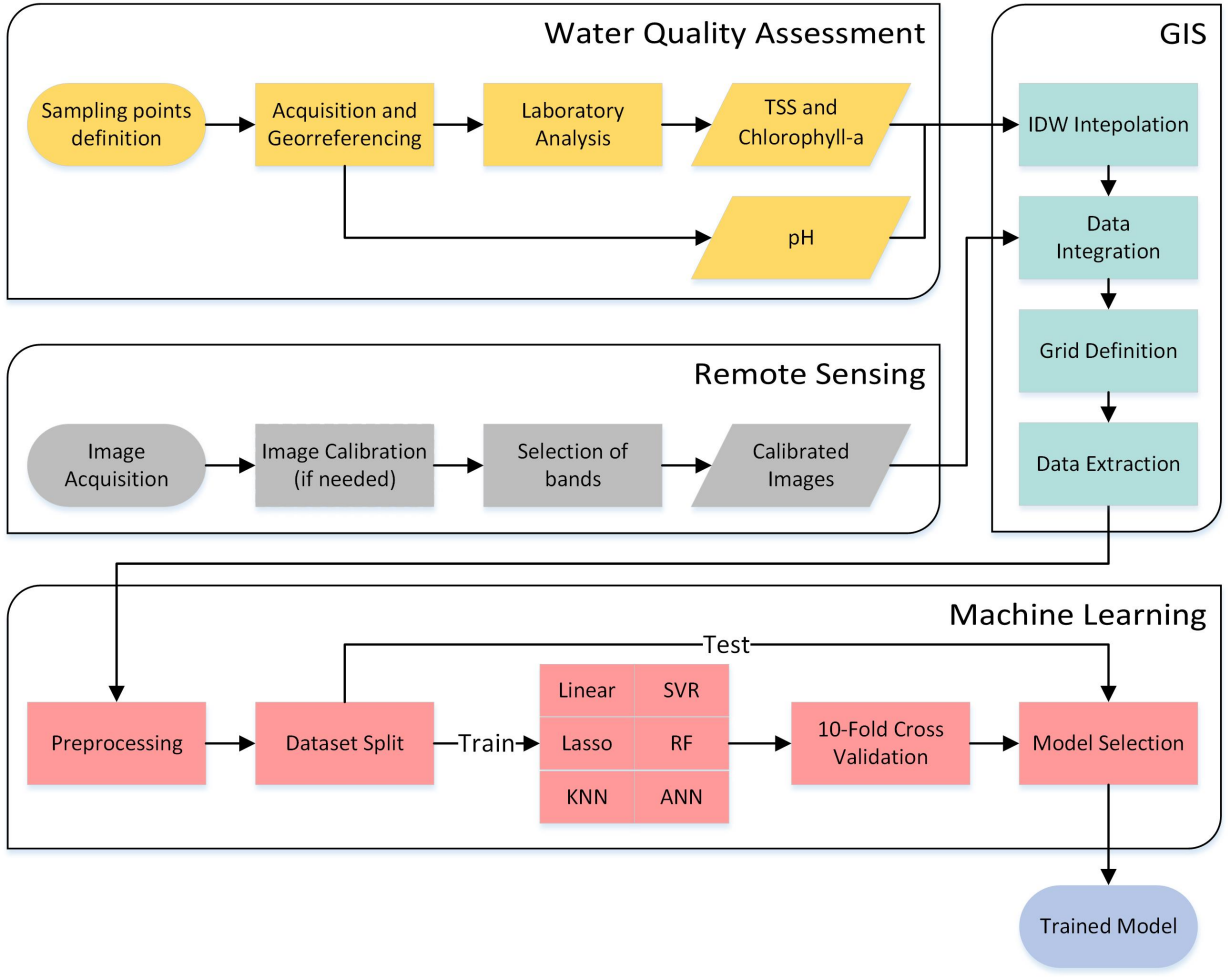
Flowchart of the proposed method.

In this section, we present in detail the proposed method for prediction of chlorophyll-a and TSS using ML techniques. Figure 1 graphically represents the methodology, and this image includes the steps necessary, including field planning, sample collection, laboratory analysis, image acquisition and processing, georeferencing, interpolation, and ML training.

For better understanding of the methodology, the flowchart (Figure 1) is divided into four macro areas of expertise illustrated as bounding boxes with the respective names. These areas of expertise are: water quality assessment (field planning, sample acquisition, and laboratory analysis); remote sensing (image acquisition and calibration); Geographic Information System (GIS) (interpolation, data integration. and extraction); and machine learning (data are preprocessed; regression models are trained, cross-validated, and chosen).

Each of these bounding boxes is explained in the following subsections.

### 2.1. Water Quality Assessment

The person using this methodology should define strategic positions for water sample collection, so the data are representative of both chlorophyll-a and TSS. Water samples must be collected at about 0.2 m in depth for further laboratory analysis. The pH of each sample must be measured with a multiparameter water quality probe. The geospatial coordinates of each selected point must be recorded for posterior correlation with data acquired from remote sensing.

The collected samples must be subject to laboratory analysis to define chlorophyll-a and TSS amounts. The gravimetric method [21] is considered for TSS, whereas the spectrophotometric method [22] is used for chlorophyll-a concentration measures in each of the water samples.

### 2.2. Remote Sensing

For the remote sensing step of this method, two types of data sources can be used: Sentinel-2 satellite or a camera/sensor coupled to a UAV. The choice of the type of sensor used should consider aspects such as the size of the study area (small water bodies may not be mapped correctly by satellite images, for example) and the spatial resolution required for the research (UAVs have better resolution, although they demand more work and cost in the image acquisition and usually do not include multispectral channels).

The MultiSpectral Instrument (MSI) sensor onboard the Sentinel-2 (S2) satellite has 13 spectral bands from visible to infrared (443 nm–2190 nm). We adopted Sentinel-2 in this methodology because, compared to Landsat 8 (L8), it has a higher spatial resolution (10 and 20 m in comparison with the 30 m of L8), a shorter revisit period (from the 16 days of L8 to 5 days for S2), and three additional bands in the red edge region (703, 740, and 783 nm) [23], which makes it more suitable for vegetation and limnological studies [6,9].

Sentinel-2 images can be acquired for free from websites like Copernicus Open Access Hub and USGS Earth Explorer. We highlight that images are available to users as Top-Of-Atmosphere (TOA) reflectance data. To be comparable with water quality data, these images must be corrected for surface reflectance data.

If UAV images are adopted for the research, a sensor (camera) that has channels in the visible region (Red, Green, and Blue (RGB)) and Near-Infrared (NIR) must be used in the drone for area mapping. UAV imaging over the study area is performed under a setup taking into account the intended spatial resolution and region-of-interest, such as altitude, flight speed, frame overlapping, etc. At this time, Ground Control Points (GCPs) must be also established in the flight coverage area, so that their positions can be later used in georeferencing of the images obtained. For higher spatial precision, the GCPs can be tracked using GNSS (Global Navigation Satellite System), based on the Real-Time Kinematics (RTK) method.

The final product of the remote sensing steps is a georeferenced orthomosaic. To obtain one, we need to process the images taken by the UAV’s camera, and this can be performed by photogrammetry software, through the Structure from Motion (SfM) technique [24], which is capable of recreating the camera’s position in the 3D space for every photograph captured, aligning them automatically. The surveyed GCPs are used in this workflow for georeferencing and scaling of the final products [25].

We highlight that we assumed in this methodology that for images collected by UAV at low altitudes (up to 200 m), it was not necessary to perform the atmospheric correction. The atmospheric effects in the visible and NIR zones at this height are very small and almost uniform along the considered spectral range. In order to retain sufficient spatial correspondence between remote sensing or UAV data and samples collected in fieldwork, it is mandatory to acquire images at times as close as possible to the field sampling.

Seeking to integrate the two data collection options (images) proposed for this stage of the methodology, only spectral bands in the visible (RGB) and NIR regions that were present in the two sources were selected for the chlorophyll-a and TSS prediction models. The red channel was excluded because there are several low-cost sensors (cameras) available on the market, used in UAVs, that are modified to present the NIR channel in place of the red band. Besides that, the B, G, and NIR bands are widely used for similar applications involving chlorophyll-a [6,26] and TSS [11,26,27] prediction in water bodies. For Sentinel-2, we considered Bands 2 (Blue, 0.490 μm), 3 (Green, 0.560 μm), and 8 (NIR, 0.842 μm), which have similar bandwidths to the sensors used in the UAV.

### 2.3. GIS

In this step, water quality data collected in the field and laboratory were processed using the Geographic Information System (GIS) with the purpose of increasing the available dataset. This dataset was then compiled with necessary remote sensing data for subsequent application of ML techniques.

First, interpolated maps of pH, chlorophyll-a, and TSS parameters must be generated for the study area. Although spatial interpolation methods are often used in GIS to estimate the values of the variables of interest in places where there is no information, there are no conclusions about the ideal method for application in water quality surveys [28]. The interpolation methods kriging, natural neighbor, and Inverse Distance Weighting (IDW) were tested with laboratory values, considering the leave-one-out validation technique (70%–30%). Although IDW is a simpler technique compared to more complex stochastic models, like kriging [28,29], it was the method that best described the behavior of chlorophyll-a and TSS in the data. This result was consonant with [29,30]. Therefore, for this methodology, the IDW interpolation was adopted. It is important to highlight that the researcher must pay attention to the spatial resolution necessary for the study in the interpolation process, ensuring that the product generated is compatible with the other data collected and/or processed. The IDW interpolation map should be generated in the same spatial resolution as the imagery collected; by doing this, it is possible to obtain a specific value for TSS, chlorophyll-a, pH, and reflectance for each of the pixels in the image.

After interpolation, all rasters (processed remote sensing images and spatialized water quality maps) must be integrated and their standardization confirmed, such as pixel size (spatial resolution), reference system, projection, etc.

With this method, it is possible to increase the available dataset because it converts the point data collected in the field into an interpolated surface and, therefore, data in the raster format where each pixel contains information regarding each selected parameter. Therefore, it is necessary to establish a new set of sample points that have the necessary size for the purpose of the research in question. We propose to generate a grid of points with fixed spacing between them, for example 5 to 10 pixels at a distance depending on the spatial resolution of the image used (satellite or UAV).

The last step then would be to extract numerical data from these images to create a dataset. We emphasize that to be effective and to represent the chlorophyll-a and suspended solids present in the water accurately, the data obtained from the processed images ideally should represent only the spectral response of the water, with the least noise possible. Any deviations from this ideal conditions could introduce errors that would propagate to the next steps and invalidate the results. Interferences in the reflectance of water pixel values can occur mainly at the margins, with aggregated responses from more than one target (water and margin) or shadows (mainly from trees) and even the bottom of the lake in cases where the margin is shallow. It is interesting to erode the grid points to discard areas that are more susceptible to these problems. The erosion size may be altered according to the size of the study area and the spatial resolution of the image used, varying from 5 to 50 m, for example.

Having performed this process, for each point, the following information is extracted: pH, the three bands of the images (NIR, G, and B), chlorophyll-a, suspended solids, and the geodesic coordinates of each point. Point location information is important because aquatic environments are rarely homogeneous, and the spatial distribution of the parameters can vary according to characteristics external to the lake or dam and specific to each location, such as the use and occupation of the surrounding soil and the presence of other bodies of water with different water quality conditions (worse or better) draining into the study area.

### 2.4. Machine Learning

After the GIS phase has been executed, we need several steps where ML techniques are applied to assemble, train, and choose the best model. In this paper, there were experiments considering the following techniques: linear regression, LASSO, Support Vector Regression (SVR), K Nearest Neighbors (KNN), Random Forest (RF), and Artificial Neural Networks (ANN). The algorithmthat runs the experiments was developed in Python, using many code libraries, including scikit-learn, TensorFlow, and scipy-stats. Code and data available at https://github.com/lucaskup/TSS_ChlorophyllA_Prediction.

The algorithms used here belong to the class of supervised learning, and they require an annotated dataset with many known input-output pairs to be used for training (with the exception of KNN, which essentially does not need a training phase, but could have a data structure fitting phase that is analogous to the learning phase). Another characteristic is that we are using the techniques to approximate numerical values, not categorical ones. Essentially, the algorithms are trying to approximate a mapping function from a given feature space to a target space.

Reflectance, pH values, positional information, chlorophyll-a, and TSS concentration data all come to the ML learning phase of the proposed method in different formats and scales. This could be a problem for some learners, which is why a preprocessing was performed, a common step in ML or data science studies [31]. In this step, data underwent two processes, standardization and feature encoding. Usually, ML provides better results and/or faster convergence with input data limited from −1 to 1 and with categorical features encoded in binary features [18,32]. The feature encoding technique used in this paper was the one-hot encoding, also known in statistics as dummy variables, where a categorical feature is represented as a vector of binary digits in which only one bit is active in each of the observations to represent the category [33].

Despite the fact that some of the ML algorithms used (such as RF) can receive input data in arbitrary scales, we chose to consider data normalization for all the algorithms. Since it should not affect some learners and it is a necessity for others, it was decided to do this so that the processing pipeline for all ML algorithms was shared.

**Figure 2:**
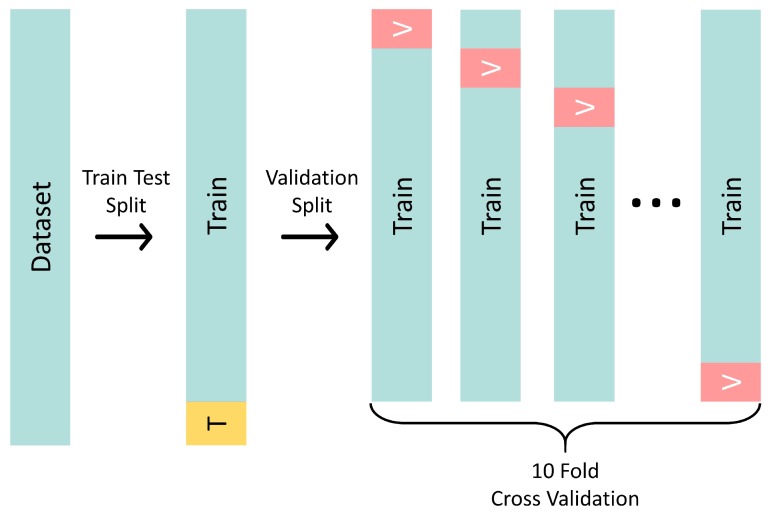
Train-test cross-validation split methodology used in this paper. The first operation applied is a train-test split that randomly separates 90% of the data as training and 10% for testing. The second operation is a validation split that uses only the train split of the previous step and produces ten different folds of training and validation splits.

The second step consisted of a train-test dataset split. This split involved randomly choosing 90% of the rows as a training set and 10% as a test set (see Figure 2, Train-Test Split arrow); this was done to access the model’s ability to generalize to unseen data [34]. The training and test set were forwarded respectively to the training process, where the process of parameter tuning was done and the model was assessed in a process of cross-validation, and to the model selection step, which is detailed in the next paragraphs. It is important to keep the test split completely out of this process to avoid bias in the trained model [35].

Knowing that the quality of regression is hard to assess [36], two different strategies for model selection were tested, the holdout and K-fold cross-validation. Although holdout is both simpler and faster, we chose to apply the K-fold cross-validation because it provides more consistent results as it runs metrics in the entire dataset after the *k* runs, so we avoided biases that a single split on the dataset could produce, providing more confidence in the results [37]. This technique divided the dataset into *k* folds of randomly chosen observations. After that, a model was trained for each of the *k* possible training test splits, and the reported values for $R^{2}$ and MSE were the average of these *k* runs. For this paper, the chosen value for *k* was 10, as this is a typical choice [38]. This is depicted in Figure 2 by the Validation Split arrow.

Even knowing that cross-validation is a standard way of evaluating learners, it is not immune to downsides. Running many configurations of algorithms on the same dataset for cross-validation can produce good results by chance [39], so in this paper, we also implemented a step of testing in our algorithm to see how it behaved when compared to the cross-validated results. When an algorithm achieves a satisfactory result in training and a poor result in testing, this is a phenomenon called overfitting [40], and the structure of cross-validation plus testing aims to avoid or at least identify this problem. The testing of the algorithm used the fraction of the dataset marked with a capital “T” in Figure 2.

It is important to emphasize that both of the dataset splits introduced in the previous paragraphs were performed only once for all the experiments with the six learners; therefore, the same partitioning was considered in all ML experiments. By doing this, we avoided a bias that could be introduced by different random splits [41].

Since all the algorithms were independent of each other, it was trivial to run the learning processes in parallel or even to implement each of the K-folds in parallel. However, in our experiments, such parallelization was not necessary since all but the ANN models were trained in a negligible amount of time.

(1) $$MSE\left(y,\hat{y}\right)=\frac{1}{n}\sum_{i=0}^{n-1}{\left(y_{i}-\hat{y_{i}}\right)}^{2}$$

(2) $$R^{2}\left(y,\hat{y}\right)=1-\frac{\sum_{i=0}^{n-1}{\left(y_{i}-\hat{y_{i}}\right)}^{2}}{\sum_{j=0}^{n-1}{\left(y_{j}-\bar{y}\right)}^{2}}$$

All the K-fold models were evaluated using $R^{2}$ and MSE metrics, which are defined in Equations (2) and (1), respectively. In those equations, *y* and $\hat{y}$ are the vectors with *n* observations from the IDW interpolation (considered as the ground truth) and predicted values, respectively, while $\bar{y}$ is the average value for the *y* vector.

MSE is a measure of the mean error between observed and predicted values. It serves as the loss function to be minimized by ANN. $R^{2}$, on the other hand, is assessed one time for each of the K-folds in the models’ testing phase. It compares how the variation in the data is captured in the predictions, being one of the best possible values for this metric [34].

The preprocessed dataset was submitted for the 10-fold cross-validation split train-test under all the six ML algorithms explored in this paper. Those algorithms offer a range of both linear and non-linear techniques, and although some of these algorithms have the property of being general function approximators given enough data [42,43,44], different algorithms can have better results on different datasets [45,46].

In the model selection step, all six learners were trained one extra time with the full training set, presented in Figure 2 with the “Train” label before the Validation Split. $R^{2}$ and MSE were then computed regarding the test set one time. If those results were not coherent with the cross-validated ones, it meant the models did not generalize well to unseen data, and as a consequence, there must be a backtrack to the “Dataset Split” step before any further attempt to tune the model parameters. This was done to avoid bias in the trained models and assure that no information from the test set was used for training, even if it was indirectly.

If one of the models performed better than the others, it was pretty straightforward to choose the best learner for the problem. Otherwise, if the results of different algorithms were close, it may be necessary to apply the Friedman statistical test [47] to see if there were differences in the cross-validation results.

The result of the ML sub-process was the model that best fit the behavior of chlorophyll-a and TSS according to the metrics chosen. The chosen model was ready for further inference in the production environment. Given as premise that the data collected in previous steps were representative of the monitored area, it was possible to standardize and feature encode new vectors of observations and run this through the model to create new inferences of chlorophyll-a and TSS without laboratory analysis.

## 3. Case Studies

**Figure 3:**
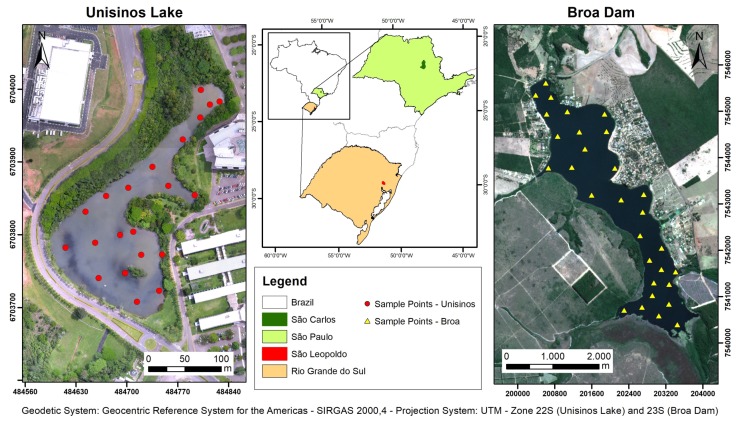
Study areas.

Aiming to validate the proposed approach, two water bodies in different regions of Brazil were visited to extract information about water quality. The regions differed in the type, size, usage, and climate conditions. One of the areas was a small artificial lake located at Unisinos University, in São Leopoldo city, Rio Grande do Sul state, in the south of the country. The other area was Broa’s Reservoir, a dam located in Itirapina city, São Paulo state, in the southeast of the country. Figure 3 shows the location map of the study areas.

For Unisinos Lake, a flight with a UAV was adopted because the total area was relatively small (0.025 km^2^), and its monitoring was unfeasible by satellite images.

Despite its small size, Unisinos Lake has a depth of 4 m (at its center) and is located at the lowest altitude of the campus. Furthermore, since it is composed of rainwater drainage collected in the university, it includes several inorganic and organic components in the form of suspended solids or organic matter from rainwater runoff [48]. The climate of the region is characterized as a humid subtropical zone with abundant precipitation distributed throughout the year.

In the field collection step, 21 sampling points were defined over Unisinos Lake (Figure 3), and two surveys were performed: one in March 2016 and the other in March 2017. In each survey, the UAV overflight was carried out on the same day. The UAV used to take the images was the SenseFly, Swinglet CAM model with the Canon ELPH 110HS camera with 16 megapixel resolution aboard. As the camera was factory modified to capture the NIR band instead of the red band, the bands obtained were B, G, and NIR. The UAV image processing was performed in the PIX4D (https://www.pix4d.com) software, Version 2.1, which generated as a final product an orthorectified and georeferenced image with a spatial resolution of 5 cm. The reference system used was SIRGAS 2000 (Geocentric Reference System for the Americas), in the Universal Transverse Mercator (UTM) projection, 22S zone.

To allow a per-pixel comparison between the measured and the estimated values, the in situ data were meshed to produce a grid of points corresponding to the spatial image. In addition, we adopted a distance of five meters to erode the grid of points to avoid interference from the margin, shadows, and even the bottom of the lake in the shallower margins. Data were extracted from each of the points in the grid. Thus, the dataset size available for the application of the machine learning techniques for Unisinos Lake went from 42 (21 points in two collections) to 35,028 data (17,014 points).

Since the Broa Reservoir has an area of 6.8 km^2^ and maximum dimensions of 8 km long and 2 km wide, it was possible to use Sentinel-2 image data. This environment is deeper than Unisinos Lake with an average depth of 3 m and a maximum of 12 m in the area near the dam.

The Broa dam was built for electricity production, but it is also used for recreational activities, sports, and amateur fishing. The reservoir is located in an area with different land uses, such as occupation by residences, reforestation, and crops, and it is formed mainly by three rivers (located in the southern part; see Figure 3), one of which has worse water quality because it receives the effluent from the city’s sewage treatment station. Although the climate is humid subtropical, unlike the Unisinos region, this one has two well-divided seasons: rainy (summer) and dry (winter).

At Broa dam, the field sampling was performed on three occasions during 2018: January (rainy season), May (intermediary), and July (dry season). Samples were collected at 30 points distributed in the dam area (Figure 3).

The Sentinel-2 images were acquired from the USGS Earth Explorer website with a difference of three to seven days between image and field collection. It is important to notice that, due to the very small dynamics of the dam waters, this time lag was not decisive for the analysis. Atmospheric correction of TOA reflectance to surface reflectance data was performed using the Sentinel-2 Atmospheric Correction (Sen2Cor) processor, available in the SNAP 6.0 software.

The grid produced from the measured data considered a spacing of five pixels between each new point in the set, that is a distance of 60 m. Considering the spatial resolution of the Sentinel image and that the dam has large extensions of shallow areas on the margins, we eroded the grid at 50 m to ensure that only data containing pixels of pure water would be computed. From this process, the number of points in the area increased from 30 to 1311, resulting in a total of 3933 data for the machine learning techniques.

## 4. Results

The following subsections are organized to present the results of each experimental step of this work sequentially.

### 4.1. Water Quality and GIS Results

The steps of data collection in the field, laboratory analysis, and finally, GIS techniques were fundamental to produce results that were used to generate regression models to predict both chlorophyll-a and TSS in the two areas of study. Since the data used in the ML algorithms passed through a process of interpolation, it is essential to assert that the interpolation did not change the general characteristics of field/laboratory data. Table 1 provides descriptive statistics that can be used to compare both field and IDW data. Modes were obtained by separating the distributions into 20 bins.

**Figure 4:**
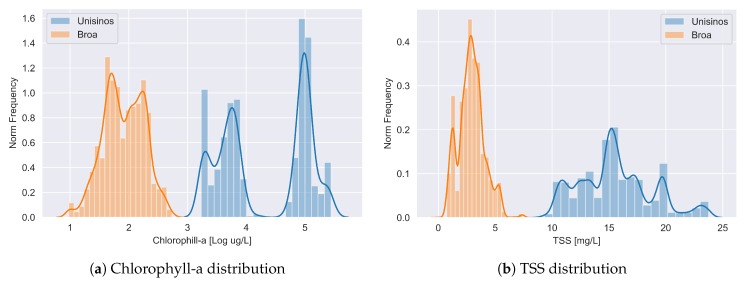
Dependent variables’ distribution from both study areas considering the data post-IDW interpolation.

Observing Table 1, is possible to see that, although the sample size increased by two and three orders of magnitude (respectively for Broa and Unisinos), the descriptive statistics such as the mean, standard deviations, and median of chlorophyll-a and TSS stayed roughly the same. The most affected value was for chlorophyll-a concentration for Unisinos, more specifically its median. To better understand this behavior, we need to look at Figure 4a: this distribution appeared to be bimodal. Therefore, since the median and mean values were not representative considering the distribution, this was not a concern. On the other hand, the two modes (better descriptive statistics for bimodal distributions) presented in the table for chlorophyll-a did not appear to have relevant changes.

The differences between the two areas of study were evident when comparing the concentrations of chlorophyll-a and TSS in these environments, where Unisinos Lake presented much higher concentrations. In addition to the values, it was also necessary to analyze the distribution of the studied variables in the two places, presented in Figure 4.

The Unisinos Lake collections were made in two consecutive years (2016 and 2017), and although they were at the same time of the year (March), the climatic conditions of the period were different. This bimodal distribution of chlorophyll-a indicated that this parameter was more present in one year than in the next. Furthermore, Unisinos data had more variance, whereas Broa’s were more concentrated around the mean value. Regarding TSS, Unisinos’ distribution was also widespread, but there was no sign of bimodal behavior; see Figure 4b).

After the interpolation, the data were sampled from the raster file in a regular grid to create a tabular version of the data. The grid and interpolation were done to allow a representative sampling of the whole water body while also creating a dataset with fewer rows, which was easier to handle in the ML phase. This tabular dataset was then forwarded for the next step in the methodology.

### 4.2. Machine Learning Results

The feature space of the regression problem was then defined by the bands blue, green, and NIR plus positional information and interpolated pH, as well as the target space, interpolated chlorophyll-a, and TSS. These data was compiled in a different dataset for each of the study cases.

The second step in data pre-processing was to deal with georeferencing data. As was mentioned in the previous sections, some water bodies have characteristics that change depending on spatial location. In the Broa dam, for example, there are some water sources from sewage treatment stations in the southern area. We wanted to provide some spatial features to the model that would allow inferring those differences, and at the same time, we wanted to avoid providing all spatial information that was used in the IDW interpolation to avoid biasing the model. This scenario was approached by separating the spatial coordinates of the image into four quadrants and providing the quadrant number, instead of providing detailed x-y coordinates for the model learners. Experiments showed that overfitting happened if positional coordinates were used directly in the feature space.

The same train-test split of the dataset was used on all six algorithms to assess and evaluate the ML algorithms. From the train split, a K-fold cross-validation was performed, and the mean values for $R^{2}$ and MSE were computed and are presented in the Table 2. By analyzing this table, we can see that linear regression, LASSO, and SVR reached the lowest $R^{2}$ and higher MSE values, whereas KNN, RF, and ANN reached the highest values for $R^{2}$ and the lowest values for MSE on the algorithms tested. The highest values for $R^{2}$ in each trial are presented in boldface.

**Figure 5:**
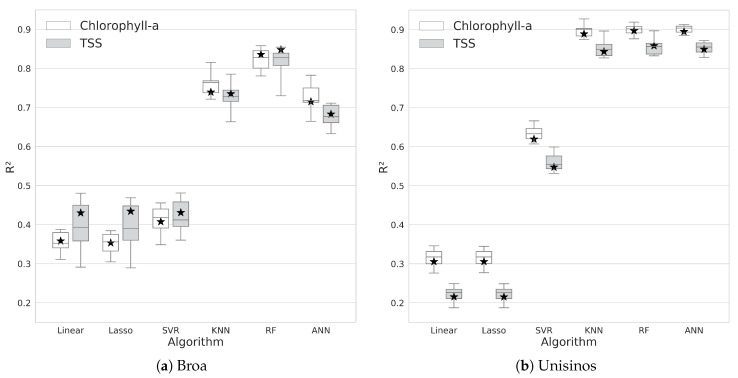
Boxplot representing the $R^{2}$ metric obtained on the cross-validation; the metric on the test set is marked with a star.

After the cross-validation step, the whole train split was used for training, and the model was executed against the test set (in Figure 2, the whole train and the whole test split are shown after the Train-Test Split arrow). Both the training cross-validation results and the test run were compiled into the graph in Figure 5 and are denoted as boxplots and stars, respectively. We can see that each of the test runs remained within the results of the cross-validation step. Among the three best algorithms, only the RF test result was not between the first and third quartile range considering the TSS result on Broa dam.

Linear and LASSO regressions achieved a median value for $R^{2}$ under 0.4 on Broa and under 0.3 on Unisinos regarding TSS. Those values indicated that the methods were not able to learn the behavior of the data correctly. Since they created linear transformations between dependent and independent variables and nonlinear methods like RF performed better, we could assume that the correlation between independent and dependent variables had some nonlinear behavior in the feature space.

Regarding the models created for the Unisinos study case, KNN, RF, and ANN achieved an $R^{2}$ consistently above 0.8 with a small variance in the results (see the boxplot sizes in Figure 5b). By inspecting this graph alone, it was not possible to decide which of these three algorithms performed better for Unisinos. Table 2 shows that ANN performed a little better on chlorophyll-a, whereas RF outperformed other learners on TSS, but all those differences were marginal.

Beyond visual inspection of Figure 5, Friedman tests [47] were performed on the cross-validation results for the KNN, RF, and ANN algorithms. The statistical tests were run on all four combinations of the study case/studied variable: Broa-chlorophyll-a, Broa-TSS, Unisinos-chlorophyll-a, and Unisinos-TSS. In each of these cases, the results for K-fold cross-validation of the algorithms KNN, RF, and ANN were considered in the statistical test. For Broa, the null hypothesis was rejected in both cases, with p=0.0004 and p=0.0003 for chlorophyll-a and TSS, respectively. Usually, a post-hoc test is necessary to identify which algorithm is better, but in this specific case, by inspecting Figure 5a, it is trivial to indicate that in this specific scenario, RF outperformed the other algorithms. For the Unisinos study case, the null hypothesis could not be rejected, p=0.27, and p=0.15 for chlorophyll-a and TSS, respectively. No significant difference between the algorithms was detected in this particular study.

### 4.3. Application of the Trained Model

Since the random forest algorithm provided the best results consistently among the trained models, being the best learner for the Broa study case and being among the best for Unisinos, we present in this subsection an application of these trained models.

**Figure 6:**
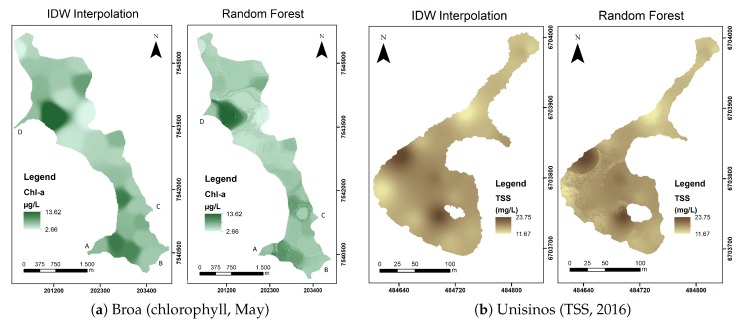
Comparison between the maps generated by the random forest algorithm and through IDW interpolation.

We applied the RF algorithms to all processed images of the study areas in order to observe whether the predicted maps generated for each variable presented a spatial distribution consistent with the expected result (maps with sampling data interpolated). Figure 6 shows two examples of this comparison, one for Broa considering the parameter chlorophyll-a and the collection for May 2018 and another for Unisinos with TSS and the collection for the year 2016.

Analyzing Figure 6, it is possible to state that the maps generated by the RF algorithms had similar spatial distributions comparing to the IDW interpolation of the sampling data. There were some areas in which concentrations formed some details in the generated maps that did not appear in the original ones.

**Figure 7:**
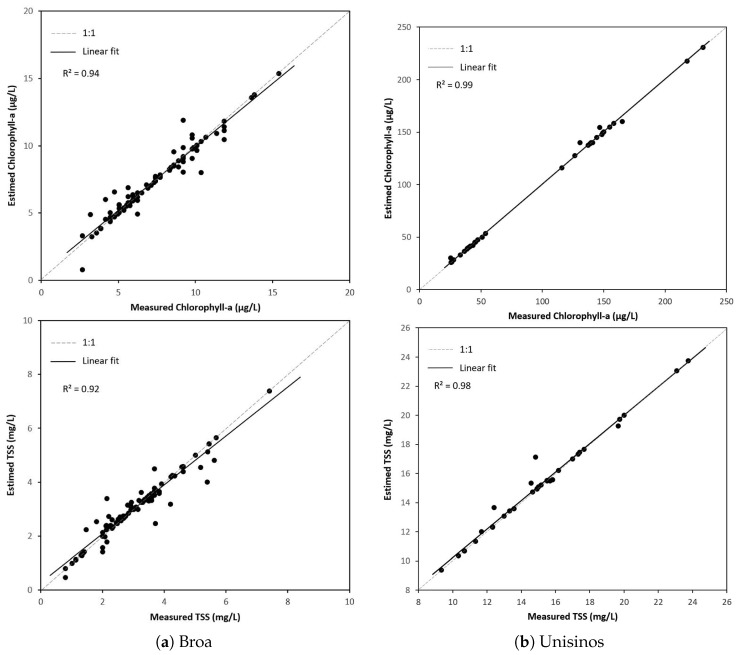
Comparison between field measurements with the random forest results.

In Figure 7, we present a scatter plot between the data points obtained through laboratory analysis and the RF results. In both cases, we see $R^{2}$ values close to one. Specifically in the Unisinos Lake study case (Figure 7b), it is evident that the laboratory values did not provided the entire range of chlorophyll-a concentration (see the lack of points in the range from 70 to 110 μg/L), but this was overcome in part by the interpolation process.

## 5. Discussion

The results achieved for both TSS and chlorophyll-a predictions reached a higher $R^{2}$ coefficient than research studies for the same region [20,27,48], as well as other similar studies over different areas [1,26]. Since the two studied regions present different environmental characteristics, these results indicated the soundness of the proposed method for water quality monitoring through remote sensing and ML techniques.

Data quality is vital for ML algorithms. The number of sample points, weather conditions, the spatial distribution of sample points, and the size of the dataset are all choices that have to be made in order to create a dataset that is representative of the studied area. In this paper, water samples were collected in different periods of the year in the same place (guaranteed through georeferencing). However, this methodological constraint may not create perfectly balanced datasets. Some imbalance in the dataset can introduce bias in the learned model; the person using the proposed method should be aware of techniques to avoid this type of problem if it arises. IDW is an efficient alternative to augment the number of samples in a dataset. It was explored in this paper, and it could enable studies in conditions that would not be viable otherwise.

The central idea behind using IDW interpolation and grid extraction of data on both study cases is to generate more data points for better training of the ML algorithms. It is crucial to keep in mind that the interpolation should not drastically change the characteristics of the dataset; the definition of the point for sample collection and laboratory analysis should be carefully picked in order to produce a representative sample from chlorophyll-a and TSS considering the properties of the water body studied. Any deviations from these requirements should trigger a warning, and the researcher may consider backtracking and choosing a new set of point for collection and laboratory analysis.

A visual analysis like the one in Figure 7 signals the precision and accuracy of the model considering the estimated values and laboratory data. However, it is noteworthy that IDW interpolation was still affecting this plot indirectly, because it provided data for the model training.

Regarding the details that appeared on the chlorophyll-a and TSS concentration maps estimated with the ML methods in Figure 6, a possible explanation for this is that the IDW interpolation equation considered only the values of each sampled point and the distance between these points to generate the spatialized surface. At the same time, RF was trained to predict variables with water reflectance data, pH, and the location quadrant in the study area; thus, it was difficult to assert which of the concentration maps was closer to the real values because both models had inherent errors.

It is also possible to observe that on the left margin, next to one of the dam’s water sources (in Figure 6a), Point D), there was a possible source of water contamination, which was identified by laboratory analysis of the sampling point located here. Comparing the two maps, it is noticed that the prediction by the RF algorithm indicated a wider distribution of this plume. This may happen because IDW spatializes the results that were collected, the spatial distribution of the values in circular regions whose sampling point is the center. The RF map, on the other hand, considers the reflectance values of each pixel in addition to the interpolated pH data and presents smoother distributions, possibly more consistent with the real situation of the water body.

The definition of the feature space and data collected in the field must be done carefully. This methodology focused on the blue, green, and NIR bands because they are often available in many satellite missions and are also not very expensive to embed in a UAV. Although the method itself should work whenever there are additional bandwidths available, in this paper, we chose to keep the number of independent variables at a minimum to create simpler and lighter models.

The results of the algorithms were analyzed considering both cross-validation and testing metrics. In the previous section, a Friedman test was conducted to analyze the degree of similarity among the algorithms in the studied dataset. For practical purposes, a visual inspection should be enough to identify the best (or at least one of the best methods). The statistical test should likely be used when there is considerable overlap in the quartiles on the boxplot. Although this is a subjective criterion, an analyst trying to replicate the results of this paper should not face further problems.

ML methods are prone to many known problems. The most common ones are the curse of dimensionality and overfitting. This paper tried to avoid those two pitfalls by encoding some best practices in the method itself. Since we limited the number of input reflectance bands, pH, and positional information, we kept the feature space to a reasonably low dimension. Overfitting can be challenging to identify and to avoid [39,40], but the method of train-test split plus cross-validation is in consonance with the best practices [46]. Although that discussion is centered on the ML phase of the methodology, its solution concerns the whole process. For instance, the cross-validation was only possible because data were densified in the GIS phase through interpolation; collecting and analyzing in the laboratory almost 4000 samples for Broa and 35,000 for Unisinos would most certainly make this work unfeasible.

There is still room for improvement in the methodology presented in this paper. In the ML phase of the methodology, only six types of learners were used, but other techniques could also benefit from this type of data. Convolutional neural networks could benefit not only from reflectance alone, but also from texture and other features learned through backpropagation.

Although TSS and chlorophyll-a could be predicted in two regions with very distinct characteristics of size, usage, and climate, new experiments may be carried out in other scenarios allowing adjusting to specific local features to achieve the best performance for each place. To allow other areas to be studied with the same method, this paper presented some flexibilities, such as: the number of sampling points; date of collection; spatial resolution of imagery used in remote sensing steps; and selection of the best algorithm in machine learning steps.

The performance of the models created by this methodology, as well as the natural behavior of chlorophyll-a and TSS concentration are subject to seasonality and study area, whereas the proposed methodology to create these models was not. This affirmation was based on the fact that no input variable modeling the seasonality was considered in the proposed method. Reflectance was enough to achieve the results of this paper. Seasonality was indirectly represented in the dataset by choosing the sampling dates of interest.

## 6. Conclusions

Considering that aquatic systems are subject to degradation, monitoring TSS and chlorophyll-a is essential for sustainability and the better management of water resources. There is evidence in the literature that these parameters are optically active components and that it is possible to approximate their concentration by putting together remote sensing and machine learning.

This paper corroborated this idea and contributed with a validated method to create prediction systems for chlorophyll-a and TSS concentrations accounting for sensors of different spatial resolutions. Since the studied places had different concentrations and dynamics for chlorophyll-a and TSS, our assessment of the ML systems showed the robustness of the method for different types of water bodies.

The results presented were promising, allowing predicting chlorophyll-a and suspended solids, as well as their temporal and spatial variations. The methodology could be extended for future systematic mapping in a secure fashion for the same investigated places. For different scenarios, new experiments could be performed in order to fit specific environmental characteristics to achieve the best performance of the method, improving the analysis and assisting parties involved in the management and control of water resources.

## Figures and Tables

**Table 1:** Descriptive statistics of the field data and post IDW interpolation.

		Field Data		IDW	
		Broa	Unisinos	Broa	Unisinos
	Sample Size	90	42	3933	35,028
Chlorophyll-a (μg/L)	Mean	7.35	94.30	7.18	96.88
	Median	7.20	67.52	6.71	91.65
	Mode	6–10	50–150	5.5–10	44–137
	Standard Deviation	2.66	59.04	2.46	62.12
Total Suspended Solids (mg/L)	Mean	3.05	14.93	2.92	15.56
	Median	2.92	14.92	2.91	15.34
	Mode	3	15	3	16
	Standard Deviation	1.26	3.29	1.12	3.19
pH	Mean	5.70	8.67	5.69	8.89
	Median	5.84	8.85	5.81	9.15
	Standard Deviation	0.55	0.82	0.53	0.68

**Table 2:** Mean values for the evaluated metrics in the cross-validation step. The highest values for $R^{2}$ are presented in boldface.

		Unisinos		Broa	
		Chl-a	TSS	Chl-a	TSS
Linear regression	$R^{2}$	0.31521	0.22380	0.35389	0.39648
	MSE	0.03642	0.04681	0.01642	0.01345
LASSO	$R^{2}$	0.31517	0.22379	0.35126	0.39457
	MSE	0.03643	0.04681	0.01649	0.01349
KNN	$R^{2}$	0.89644	0.85111	0.76172	0.72900
	MSE	0.00549	0.00895	0.00607	0.00602
SVR	$R^{2}$	0.63457	0.56024	0.41329	0.42219
	MSE	0.01942	0.02650	0.01491	0.01290
RF	$R^{2}$	0.90012	**0.85603**	**0.82338**	**0.81371**
	MSE	0.00531	0.00867	0.00450	0.00415
ANN	$R^{2}$	**0.90138**	0.85371	0.72573	0.67819
	MSE	0.00524	0.00882	0.00700	0.00719
